# Mitochondrial metabolic remodeling in response to genetic and environmental perturbations

**DOI:** 10.1002/wsbm.1334

**Published:** 2016-05-19

**Authors:** Kate E.R. Hollinshead, Daniel A. Tennant

**Affiliations:** ^1^ Institute of Metabolism and Systems Research, College of Medical and Dental SciencesUniversity of BirminghamBirminghamUK

## Abstract

Mitochondria are metabolic hubs within mammalian cells and demonstrate significant metabolic plasticity. In oxygenated environments with ample carbohydrate, amino acid, and lipid sources, they are able to use the tricarboxylic acid cycle for the production of anabolic metabolites and ATP. However, in conditions where oxygen becomes limiting for oxidative phosphorylation, they can rapidly signal to increase cytosolic glycolytic ATP production, while awaiting hypoxia‐induced changes in the proteome mediated by the activity of transcription factors such as hypoxia‐inducible factor 1. Hypoxia is a well‐described phenotype of most cancers, driving many aspects of malignancy. Improving our understanding of how mitochondria change their metabolism in response to this stimulus may therefore elicit the design of new selective therapies. Many of the recent advances in our understanding of mitochondrial metabolic plasticity have been acquired through investigations of cancer‐associated mutations in metabolic enzymes, including succinate dehydrogenase, fumarate hydratase, and isocitrate dehydrogenase. This review will describe how metabolic perturbations induced by hypoxia and mutations in these enzymes have informed our knowledge in the control of mitochondrial metabolism, and will examine what this may mean for the biology of the cancers in which these mutations are observed. *WIREs Syst Biol Med* 2016, 8:272–285. doi: 10.1002/wsbm.1334

For further resources related to this article, please visit the WIREs website.

## INTRODUCTION

Mitochondria are a ubiquitous feature of eukaryotic cells, thought to have been incorporated as a core component of our cellular machinery at approximately the same time as the increase in atmospheric oxygen levels, around 1.5 billion years ago.^1^ They are hypothesized to be the foundation of many aspects of metazoan phenotype, including the ability to differentiate and our considerable metabolic plasticity. Mitochondria are also unique within the eukaryotic cell, consisting of a double lipid bilayer, a specific lipid component (cardiolipin) not otherwise found in the cell, and their own DNA. As metabolic hubs of the cell, mitochondria integrate the use of diverse carbon sources, including sugars and their downstream metabolites, lipids, amino acids, and ketone bodies for the generation of cellular energy (ATP). They are also central to the conversion of one carbon source into another, permitting the synthesis of lipids from sugars and glucose from amino acids. Without them, cells would be forced to rely on exogenous nutrient sources for processes such as cell repair and proliferation.

The function of mammalian mitochondria is greatly dependent on an oxygenated microenvironment and a highly regulated complement of metabolic enzymes, some of which are unique within the cell. In the early 1900s, Dr Otto Warburg made the observation that cancer cells produce significant lactate in the presence of oxygen, which led him to the assertion that mitochondrial dysfunction was a root cause of all cancers.^2^, ^3^ Although this was later shown not to be the case as a generalized mechanism, it has not escaped the attention of cancer biologists more recently that mitochondrial dysfunction is often observed in cancer.^4^, ^5^, ^6^ However, the role of this dysfunction—whether a driver, a necessary supporter, or just a side act—is not always clear. This review will outline from a cancer perspective how mitochondrial function is known to be affected by oxygen tension, and the effect of mutations in some of the metabolic enzymes within and associated with the mitochondria that have been shown to play a role in the formation or phenotype of some cancers.

## HYPOXIA AND MITOCHONDRIAL FUNCTION

**Figure 1:**
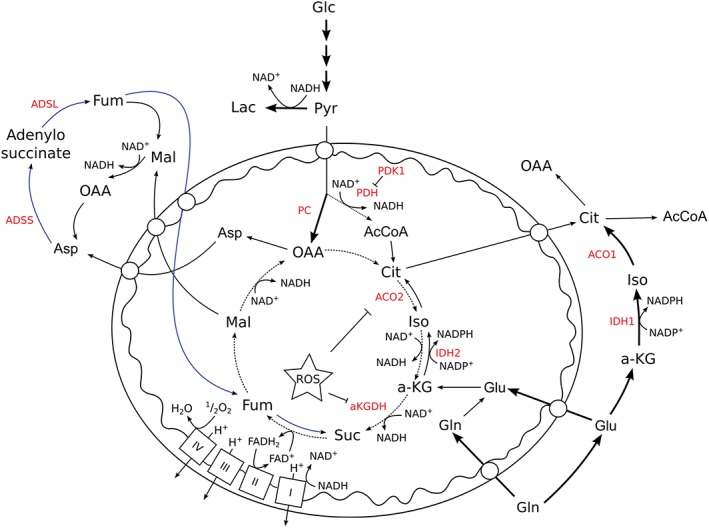
Hypoxia‐induced changes in mitochondrial metabolism. Changes that have been described to occur in human mitochondria under hypoxia are shown on a background of normoxic metabolism. Hypoxia‐induced changes in pathway use are shown as thicker lines, while the reversal of succinate dehydrogenase (SDH) activity is shown as a blue line. Metabolic enzymes are shown in red. Abbreviations: AcCoA, acetyl CoA; ACO1, cytosolic aconitase; ACO2, mitochondrial aconitase; ADSS, adenylosuccinate synthetase; ADSL, adenylosuccinate lyase; a‐KG, α‐ketoglutarate; a‐KGDH, α‐ketoglutarate dehydrogenase; Asp, aspartate; Cit, citrate; FAD^+^, flavin adenine dinucleotide; FADH_2_, reduced flavin adenine dinucleotide; Fum, fumarate; Glc, glucose; Gln, glutamine; Glu, glutamate; IDH1, cytosolic isocitrate dehydrogenase; IDH2, mitochondrial isocitrate dehydrogenase; Lac, lactate; LDH, lactate dehydrogenase; Mal, malate; NAD^+^, nicotinamide adenine dinucleotide; NADH, reduced nicotinamide adenine dinucleotide; OAA, oxaloacetate; PC, pyruvate carboxylase; PDH, pyruvate dehydrogenase; PDK1, pyruvate dehydrogenase kinase 1; Pyr, pyruvate; ROS, reactive oxygen species; Suc, succinate.

As tumors grow from a single transformed cell into a cell mass, they create a significant demand for glucose and oxygen that outweighs supply. The partial pressure of oxygen therefore decreases within the tumor, resulting in a reduced ability of cells to produce ATP through oxidative phosphorylation. In turn, the repression of respiration on glycolysis is lost, and glycolytic ATP production increases to compensate.^7^ Decreased respiration also results in a reduction in the rate of NADH oxidation by complex I of the respiratory chain, leading to an increase in the NADH:NAD^+^ ratio in the mitochondria.^8^, ^9^ This increase inhibits the reducing potential of the cytosolic NADH produced in glycolysis from being transferred into the mitochondria through the malate–aspartate shuttle. As a result, the NADH must be oxidized in the cytosol to permit continued ATP production through glycolysis by the reduction of pyruvate to lactate. Without any compensatory steps, the increase in the NADH:NAD^+^ ratio in the mitochondria means that in hypoxia, the NADH‐producing reactions of the tricarboxylic acid (TCA) cycle are inhibited (Figure 1), reducing flux through the pathway, and thereby inhibiting oxidative anabolic metabolism from mitochondrial carbon sources. As a result, cells would be forced into a nonproliferative, energy‐sparing phenotype—producing most ATP through glycolysis and downregulating processes that demand significant energy and macromolecules such as protein synthesis. Hypoxia has previously been shown to elicit an autophagic response,^8^, ^10^ which could supply some cellular macromolecules, but without a degree of mitochondrial remodeling, continued anabolism in these conditions is not possible.^8^ Instead, as a measure of compensation, the TCA cycle is split into a number of linear pathways under these conditions.

In diving mammals and marine invertebrates, which can survive underwater for significant periods, it has been noted that succinate accumulates in the peripheral blood while the animal is in hypoxic conditions.^11^ Indeed, the hypoxic accumulation of succinate has been observed in mammalian tissues, and has been attributed to the mitochondrial reduction of fumarate by succinate dehydrogenase (SDH) to continue NADH oxidation by complex I^12^, ^13^—a functional reversibility that has been known for over 50 years.^14^ This means that mitochondrial ATP synthesis as well as glycolysis can be maintained with reduced lactate production, and therefore with a longer period before acidosis, decreasing the need to resurface to service the oxygen debt. The fumarate reductase activity of SDH has now been described in both normal ischemic and hypoxic cancer cells, showing that this is a universal phenotype of mammalian mitochondria.^13^, ^15^ In these conditions, a source of mitochondrial fumarate is required. It has been suggested in ischemic cells that both import of aspartate and reversal of part of the TCA cycle and the use of the purine nucleotide cycle can provide sources of fumarate.^13^ The purine nucleotide cycle is an extra‐mitochondrial cycle that is required to aminate inosine monophosphate (IMP) to form adenosine monophosphate through the activities of adenylosuccinate synthetase (ADSS) and adenylosuccinate lyase (ADSL). The amine group is provided by aspartate, which as a result is converted to fumarate (Figure 1), and the AMP can be used in energy metabolism or to complete the cycle through the activity of AMP deaminase (AMPD), which evolves ammonium and IMP.

The metabolism of hypoxic mitochondria in a cellular context has also been shown to produce higher levels of reactive oxygen species (ROS) than in normoxia.^16^ Reduced complex I and III have been shown to donate electrons to available oxygen, forming the highly reactive superoxide radical, which can oxidize available proteins, lipids, and DNA in the nearest vicinity or be dismuted to hydrogen peroxide, which is able to travel further and oxidize molecules in more of the cellular space.^9^ There are a number of mitochondrial enzymes that are highly susceptible to oxidant‐mediated inactivation, including complex I, aconitase, and α‐ketoglutarate dehydrogenase (αKGDH).^17^, ^18^, ^19^, ^20^ Interestingly, the inactivation of aconitase and αKGDH has significantly different effects on mitochondrial metabolic activity. Inactivation of the former results in a loss of citrate oxidation in the TCA cycle but permits the continuation of glutamine oxidation, whereas inactivation of the latter would inhibit any glutamine oxidation, but could permit other NADH‐producing reactions to continue (Figure 1). Inactivation of either enzyme therefore forms linear metabolic pathways from the TCA cycle enzymes in hypoxia.

In addition to the direct effects of hypoxia on mitochondrial metabolism, there are also indirect effects as a result of transcriptional changes induced by low oxygen. The initial growth of a cancer results in areas of low oxygen tension found throughout the tumor mass, away from the functional blood vessels. This results in the inhibition of the oxygen‐mediated repression of a small family of heterodimeric transcription factors known as hypoxia‐inducible factors (HIFs). These transcription factors can then move to the nucleus where they bind the p300/CBP [cyclic AMP‐responsive element‐binding (CREB) protein] histone acetyl transferases to decondense chromatin and transcribe their target genes.^21^ The oxygen sensors for this process are a family of three prolyl hydroxylase enzymes known as the PHDs. These enzymes form part of a larger superfamily of nonheme iron α‐ketoglutarate (αKG)‐dependent dioxygenases found in multiple phyla, and use oxygen and αKG to hydroxylate a peptidyl prolyl residue in their target proteins, resulting in the formation of succinate and CO_2._ 22 The hydroxylation of HIFα subunits causes them to be recognized and bound by the E3 ubiquitin ligase, von Hippel Lindau protein (pVHL), polyubiquitylated, and subsequently degraded by the proteasome.^23^, ^24^ The exquisite sensitivity of this process means that the half‐life of HIF1α is thought to be the shortest known of any protein.

Since its discovery, HIF1 has been shown to regulate a number of transcripts that can directly and indirectly modulate mitochondrial metabolism. Indeed, HIF1 expression in hypoxia has been shown to be central for continued viability.^25^ Perhaps the best described mitochondrial metabolic HIF1 target gene is pyruvate dehydrogenase kinase 1 (*PDK1*).^25^, ^26^ The kinase encoded by this gene alters mitochondrial metabolism through the phosphorylation and inhibition of the E1α subunit of the pyruvate dehydrogenase complex (PDC), which oxidatively decarboxylates pyruvate to form acetyl CoA, a very important donor of mitochondrial acetyl groups to not only the epigenetic machinery but also the TCA cycle. The upregulation of PDK1 and consequent inhibition of the PDC has been shown as vital in maintaining cell viability in hypoxia, through the reduction of ROS production and stabilization of ATP levels.^25^ These phenotypes could be designed to avoid activation of cellular senescence pathways in hypoxia, as suppression of the PDC by oncogene activation has recently been shown as critical in suppressing oncogene‐induced senescence (OIS).^27^ Indeed, further evidence of the use of metabolic remodeling in hypoxia to avoid ROS‐mediated senescence arises from evidence that inhibition of the increased glycogen metabolism observed in hypoxia results in senescence through increased ROS production.^28^ It is therefore likely that the inhibition of the PDC by HIF1‐induced PDK1 upregulation reduces ROS‐induced senescence in hypoxia. However, loss of pyruvate oxidation can also result in a truncated TCA cycle, in which the oxaloacetate (OAA) produced by glutamine oxidation cannot be used to produce citrate without another source of mitochondrial acetyl CoA. It is possible that the pool of acetyl CoA is maintained through the β oxidation of fatty acids in hypoxia, but if the mitochondrial aconitase is also inhibited in these conditions, through the increased production of ROS in hypoxia, this is unlikely.

Loss of PDC activity has been shown to result in a change in the citrate to αKG ratio in the cell, which has been suggested to be a prerequisite for a change in the way in which glutamine is metabolized: inducing an increase in the use of reductive carboxylation of this amino acid^29^ (Figure 1). This is a potentially important pathway in hypoxia, as pyruvate oxidation is inhibited by HIF1‐dependent upregulation of PDK1, producing a deficit of the metabolites in the proximal portion of the TCA cycle (citrate and isocitrate). Indeed, as previously suggested, a reduction in oxygen tension may also be limiting for oxidation of glutamine, making reductive carboxylation more relied upon for anabolic metabolism.^30^ The enzymes responsible for the reductive metabolism of glutamine can be found both in the mitochondrial matrix and the cytosol (ACO2 and IDH2; ACO1 and IDH1, respectively), and may be able to compensate for this deficit in these conditions.^29^, ^31^ It is therefore consistent that reductive carboxylation has been suggested as an essential pathway for cancer cell survival in hypoxia or for those with defective oxidative mitochondrial machinery.^30^, ^32^, ^33^, ^34^ Although it is theoretically possible that both pathways contribute to this reductive activity, the cytosolic compartment is much more easily accessible to exogenous glutamine than the mitochondrial matrix, as the latter requires transport of the precursor (glutamine/glutamate/αKG) into the matrix and the transport of the resulting citrate out. It is unknown how hypoxia may alter the activity of the necessary transporters, which requires further study.

Further HIF1‐dependent modification of mitochondrial metabolism in hypoxia has been reported through subunit switching of complex IV (cytochrome c oxidase, COX) of the respiratory chain. COX is a homodimer with 13 subunits per monomer, which sits within the mitochondrial inner membrane.^35^ In common with most complexes of the respiratory chain, the subunits of COX are encoded by genes in both the mitochondrial and nuclear DNA. COX4, one of the centrally situated subunits of the complex, is encoded by the nuclear genome with two paralogues: *COX4‐1* and *COX4‐2*.^36^ In both yeast (in which this subunit is known as Cox5) and some human tissues, reduction in environmental oxygen tension elicits a repression of the expression of the ‘a’ or ‘1’ subunit and an increase in expression of the ‘b’ or ‘2’ subunit.^37^, ^38^, ^39^ In cancer cells, it was shown that this switching was brought about by the HIF1‐induced expression of the COX4‐2 transcript, alongside increased expression of the mitochondrial LON protease, which degraded the COX4‐1 subunit.^37^ From studies in yeast, it is thought that expression of the ‘b’ subunit may permit a more rapid transfer of electrons to COX1 from the heme moiety.^40^, ^41^ The study by Fukuda et al. in cancer cells suggests that this may reduce mitochondrial ROS production in hypoxia.^37^ However, the general overall effect of this on hypoxic mitochondrial biology is not entirely clear, as it appears that COX4‐2 expression may only be regulated by hypoxia in a subset of tissues (lung and liver).^37^

The major hydroxylase that determines HIF1α stability is PHD2,^42^ suggesting that the majority of HIF1‐mediated changes in mitochondrial metabolism are dependent on the inhibition of PHD2 in hypoxia. However, recent studies investigating the PHD1^−/−^ mouse have shown that hypoxia may induce other changes in mitochondrial metabolic activity, independently of HIF1.^43^, ^44^ These changes have not yet been adequately studied and represent a significant gap in our knowledge of how hypoxia remodels cellular metabolism in general.

## MUTATIONS OF MITOCHONDRIAL (AND ASSOCIATED) METABOLIC ENZYMES IN CANCER

The identification of cancer‐associated mutations in the metabolic enzymes such as fumarate hydratase (FH), SDH, and isocitrate dehydrogenase (IDH) has demonstrated that altered mitochondrial metabolism is sufficient to transform some cell types.^45^ *FH* and *SDH* are *bone fide* tumor suppressor genes, inactivating mutations in which have been identified as causative in cancers such as renal clear‐cell carcinoma and paraganglioma.^46^, ^47^, ^48^, ^49^, ^50^ Mutations in *IDH1* and *IDH2* are common in a variety of diverse cancers, including adult gliomas^51^, ^52^ and acute myelogenous leukemia (AML),^53^, ^54^ but unlike *FH* and *SDH*, are genetic oncogenes. A single hit, which is both loss of function and gain of function, is only ever observed in these genes. In both gliomas and AML, mutations in *IDH* genes have been suggested as a very early, and perhaps founder mutations, although in neither disease does *IDH* mutation appear to be able to induce transformation on its own.^55^, ^56^, ^57^, ^58^ What is common to all three genes is that they are either situated directly within the mitochondrial matrix (*SDH*, *FH*, and *IDH2*) or their metabolic activity directly affects that of the mitochondrion (*IDH1*). In addition, the cancer‐associated mutations observed have all been reported to interact with the HIF system; in the case of *FH* and *SDH* mutations, these unequivocally result in HIFα stabilization, whereas in the case of *IDH1* and *IDH2*, it appears that mutation may destabilize hypoxic HIFα stabilization. In the latter case, it would appear that the biological outcome of this is not yet clear. Mutations in these enzymes therefore have a dual outcome on mitochondrial metabolic activity: they both alter HIF1 activity and they directly alter metabolism.

### Succinate Dehydrogenase

SDH is a highly conserved heterotetrameric complex encoded entirely by the nuclear genome. It is responsible for the oxidation of succinate to fumarate in the TCA cycle and the transfer of electrons to ubiquinone, transfer of which is required by complex III to pump protons. Complex II itself is unique in the respiratory chain as the only complex without proton pumping function. The complex consists of two subunits (SDHA and SDHB), which protrude into the mitochondrial matrix, and two hydrophobic subunits (SDHC and SDHD), which anchor the catalytic components to the inner mitochondrial membrane and produce the binding site for ubiquinone.^59^ Mutations in *SDHD* were the first to be identified as causative in patients with hereditary paragangliomas and later pheochromocytomas, rare neuroendocrine neoplasms of the chromaffin tissue of the adrenal medulla and the parasympathetic tissue of the head and neck paraganglioma, respectively.^50^ Since the initial discovery of *SDHD* mutations in 2000, mutations in *SDHB* and *SDHC*, and more recently, *SDHA* and *SDHAF2* (encoding the protein responsible for FAD incorporation into SDH) have been identified with the same syndrome.^47^, ^48^, ^49^, ^60^ These mutations are found as heterozygous germline mutations, which means that loss of protein function and neoplastic transformation develops as a result of loss of heterozygosity, resulting in the complete loss of enzyme function by a second ‘hit.’^61^ Inactivating mutations of SDH subunits have since been reportedly associated with other tumor types including gastrointestinal stromal tumors,^62^ renal cell carcinoma,^63^, ^64^ and neuroblastoma.^65^, ^66^ Interestingly, mutations of SDHB predispose to a more malignant and aggressive form of paraganglioma than those of the other subunits, despite the apparent similarities in both functional and downstream phenotypic consequences of this mutation. It is apparent from this clinical perspective that we do not fully understand the role of SDHB in SDH function.

SDH is one of the few enzymes in the TCA cycle that are unique to the mitochondrion, making this compartment the major source of succinate, and the only one in which it can be further metabolized within the mammalian cell. Loss of SDH function through mutation in any one of the subunits results in loss of dehydrogenase activity, succinate accumulation within the cell, and excretion into the extracellular space. The significant increase in succinate concentrations leads to the inhibition of all members of the αKG‐dependent dioxygenase family that have been reported, including histone and DNA demethylases and the PHDs. This produces a hypermethylator phenotype of the chromatin^67^ as well as a pseudohypoxic phenotype through the stabilization of HIF in the presence of oxygen.^68^ This has the significant consequence of changing mitochondrial activity to a more ‘hypoxia‐like’ state through HIF1 signaling. In addition, it has been suggested that SDH‐deficient mitochondria may generate more superoxide, resulting in additional enzyme inactivation as previously described for hypoxia.^69^

**Figure 2:**
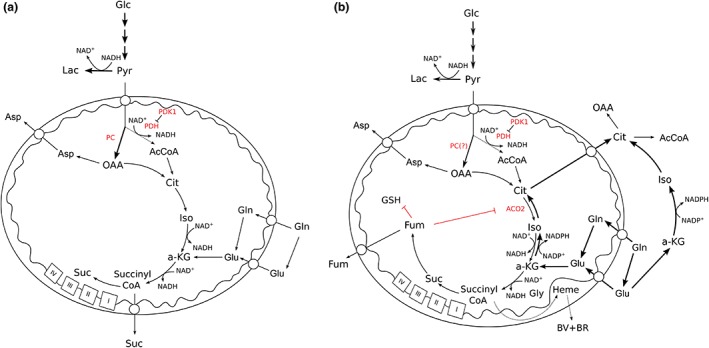
Succinate dehydrogenase (SDH) (a) and fumarate hydratase (FH) (b) mutation‐mediated alterations in mitochondrial metabolism. Metabolic pathways that have been shown to be used in cells or tumors containing these mutations are shown. Pathways that are likely to be used, but have not yet been shown are marked with ‘?’ Abbreviations: AcCoA, acetyl CoA; ACO2, mitochondrial aconitase; a‐KG, α‐ketoglutarate; Asp, aspartate; BR, bilirubin; BV, biliverdin; Cit, citrate; Fum, fumarate; Glc, glucose; Gln, glutamine; Glu, glutamate; GSH, reduced glutathione; Gly, glycine; Lac, lactate; OAA, oxaloacetate; PC, pyruvate carboxylase; PDH, pyruvate dehydrogenase; PDK1, pyruvate dehydrogenase kinase 1; Pyr, pyruvate; Suc, succinate.

Although the loss of SDH activity within the mitochondria produces another form of truncated TCA cycle (Figure 2), the significant production of succinate shows that this new TCA cycle activity is used by cells. However, until recently, the models available for interrogation of this activity were lacking. Two recent studies have described aspects of the metabolic remodeling that mitochondria undergo in the absence of Sdhb.^70^, ^71^ As would be expected from the stabilization of HIF1, Sdhb‐deficient cells were shown to have significantly inhibited pyruvate oxidation, but pyruvate was still metabolized by the mitochondria through carboxylation by pyruvate carboxylase (PC). Indeed, this was found to be critical for continued Sdhb‐deficient cell proliferation both *in vitro* and *in vivo* 70, 71 most likely due to the loss of aspartate synthesis in these cells (Figure 2)—an amino acid required for a number of key anabolic pathways including protein and nucleotide synthesis. Importantly, those cells with wild‐type SDH activity were able to tolerate siRNA‐induced PC deficiency and maintain their proliferative capability through continued synthesis of aspartate through the oxidative TCA cycle. The continued production of succinate by SDH‐deficient cells indicates the ability of their mitochondria to retain some NADH oxidative activity and therefore continued oxidative ATP production. Indeed, it appears that glutamine carbons may represent the major carbon source for the succinate produced,^70^, ^71^ suggesting that it is the activity of αKGDH that delivers significant NAD^+^ reducing activity in the absence of SDH. However, important questions remain—one of the most intriguing being whether cells and tumors bearing loss of function of SDH through mutations in other subunits show the same metabolic phenotype. This is key, as *SDHB*‐mutated tumors are the only SDH‐mutated tumors result in malignant tumors. One of the few studies to examine the differential effects of deficiencies of different SDH subunits suggested that RNAi‐mediated reduction in the expression of SDHB led to increased mitochondrial ROS production, while this was not recapitulated by similar reduction of SDHA.^72^ However, another study has suggested that SDHB deficiency does not result in detectable induction of ROS production.^73^ It therefore remains unclear as to the basis of the malignancy in SDHB‐deficient tumors, and further research is required.

### Fumarate Hydratase

*FH*, encoding the proximal downstream enzyme to SDH, is another mitochondrial tumor suppressor gene, the loss of which leads to hereditary leiomyomatosis and renal‐cell cancer (HLRCC).^46^ FH‐deficient leiomyomas (a benign neoplasm) are highly penetrant, while RCC occurs slightly less frequently.^74^ However, the RCC that develop are highly aggressive, leading to the early generation of distant metastases,^74^ similar to SDHB‐deficient tumors. Although a single gene, processing at the mitochondrion allows the protein to be distributed in both cytosolic and mitochondrial compartments.^75^

FH is responsible for the hydration of fumarate to form malate, which can then either be oxidized by malate dehydrogenase to form NADH and oxaloacetate or oxidatively decarboxylated by malic enzyme to form pyruvate and NADPH (Figure 1). Deficiencies in FH lead to high steady‐state concentrations of fumarate in the cell and surrounding extracellular space. In parallel to succinate, fumarate has been shown to competitively inhibit αKG‐dependent dioxygenase enzymes, resulting in a pseudohypoxic transcriptome through HIFα stabilization^76^ and a hypermethylator phenotype.^76^, ^77^ However, contrary to initial thoughts, now there is evidence to suggest that stabilization of HIF is not the driver of tumorigenesis in FH‐deficient tumors, and that a different candidate transcription factor—the nuclear‐related factor 2 (NRF2) pathway—may instead be involved.^78^, ^79^

In addition to the truncation of the TCA cycle and increase in reductive glutamine metabolism that occurs as a result of loss of FH function,^34^ fumarate is also a product of two other metabolic pathways (in contrast to succinate): the urea and purine nucleotide cycles as well as tyrosine metabolism. The high concentrations of fumarate produced in FH‐deficient cells have been shown to result in reversal of part of the urea cycle, producing argininosuccinate and disrupting arginine metabolism within FH‐deficient cells.^80^, ^81^ In addition, fumarate has been shown to react with enzymes and other intracellular metabolites in a process known as succination.^82^, ^83^, ^84^ This posttranslational modification has been shown to inhibit mitochondrial aconitase activity in FH‐deficient cells, resulting in further truncation of the TCA cycle^82^ (Figure 2). In addition, succination of reduced glutathione (GSH) to form succinicGSH has also been demonstrated, which results in the depletion of the intracellular antioxidant pool and senescence.^83^ Importantly, this sheds further light on the mutational hits required for an FH‐deficient lesion to become malignant, and suggests that upregulation of NRF2‐dependent antioxidant pathways, as previously described, is likely to be important to compensate for the reduced GSH pool. In mice, inactivation of cell cycle inhibitors, such as p21, has been shown to be important for tumor development in *Fh1* ^−/−^ kidneys.^83^ In addition, it is highly likely that other exposed thiol groups in mitochondrial‐associated enzymes are potential targets of succination, and therefore other metabolic perturbations may be present in these cells.

A bioinformatic analysis of *Fh1*‐deficient cells highlighted a mitochondrial metabolic ‘bypass’ required for viability.^85^ Frezza et al. showed that *Fh1*‐deficient cells continue to oxidize glutamine through αKGDH (reducing NAD^+^ for oxidation by complex I of the respiratory chain) but direct a proportion of the succinyl CoA produced by the subsequent enzymatic step of the TCA cycle, succinyl CoA ligase, through the heme biosynthetic pathway^85^ (Figure 2). This pathway, which starts and finishes in the mitochondrial matrix, uses four molecules each of succinyl CoA and glycine to produce heme, so appears to be a very efficient means of removing the excess carbon from the cell. However, Frezza et al. showed that the degradation of heme was also required, as pharmacological block or knockdown of heme oxygenase, the first enzyme in the degradation of heme, resulted in loss of cell viability.^85^ Heme degradation releases iron and CO, producing biliverdin and then bilirubin. Interestingly, the use of this pathway may not only be due to a requirement to excrete excess TCA cycle carbon. It has been noted that both biliverdin and bilirubin can act as antioxidants, capable of quenching superoxide—perhaps a useful function in cells deficient for GSH.^86^, ^87^ Although the cellular localization of heme oxygenase is not clear, it is possible that release of the CO in proximity to the respiratory chain could have consequences for continued electron flow and therefore NADH‐oxidizing activity. Importantly, the synthesis of heme also required considerable glycine (1:1 with succinyl CoA), which is likely to make cells highly dependent on the glycine synthetic pathways, controlled by serine hydroxymethyltransferase (SHMT) 1 and 2. As this part of the pathway occurs in the mitochondrial matrix, SHMT2, the matrix isozyme may be key in FH‐deficient cell function.

The bioinformatics screen performed on Fh‐deficient cells also highlighted other enzymes that may be synthetic lethal with loss of *Fh1*.^85^ Of particular note is the mention of PC, which was later shown to be synthetic lethal with loss of Sdhb,^70^, ^71^ glutaminase (Gln), which can be understood through the cells requirement for this amino acid for mitochondrial oxidative activity, and SDH. This last prediction is intriguing, but no further data are yet available to show whether this is a true synthetic lethal combination with *Fh1* deficiency.

### Isocitrate Dehydrogenase

IDH enzymes are members of the β‐decarboxylating dehydrogenase family that catalyze the oxidative decarboxylation of isocitrate to αKG and CO_2_. Mammalian cells express three IDH isoforms (1, 2, and 3) encoded by five genes (*IDH1*, *2*, *3A*/*3B*/*3G*) in the nuclear genome. IDH1 and IDH2 are reversible, highly homologous (>70%) homodimeric NADP^+^‐dependent enzymes. IDH3 is an irreversible heterotetrameric NAD^+^‐dependent enzyme, both genetically and structurally unrelated to IDH1 and IDH2. Interestingly, mutations only in *IDH1* and *IDH2* have thus far been linked to cancer. The presence of recurrent somatic point mutations in *IDH1* was first identified in 12% of all adult glioblastoma tumors by high‐throughput DNA sequencing.^51^ Deep sequencing further revealed that mutated *IDH1* and *IDH2* occur in a high proportion (>70%) of low‐grade (grade II–III) astrocytomas and oligodendrogliomas and secondary glioblastoma.^51^, ^52^, ^88^, ^89^ Since these initial reports, mutations in *IDH1* and *IDH2* have also been found in other diverse cancers including 16–17% of patients with AML,^53^ 20% of patients with angioimmunoblastic T‐cell lymphomas,^90^ and more rarely in cholangiocarcinomas,^91^ breast carcinomas,^92^ acute lymphoblastic leukemias,^93^ prostate cancer,^93^ osteosarcoma,^94^ and others.^53^, ^91^, ^92^, ^93^, ^95^, ^96^, ^97^ Interestingly, and unlike IDH mutations in glioma which are almost entirely in *IDH1*, AML have been shown to exhibit an approximately equal frequency of *IDH1* and *IDH2* mutations.^53^, ^54^

*IDH1*‐mutated gliomas have all been reported to have a single‐point mutation within the codon for Arg132. It appears that the removal of the arginine, rather than the substitution of a specific residue, is the critical feature of this mutation, although the most frequent replacement observed is a histidine residue (R132H).^51^, ^52^, ^93^, ^98^ Mutations in *IDH2* in gliomas occur at a much lower frequency (0–6%) than *IDH1* 88 in the paralogous amino acid residue—R140—but also at R172. These residues are key in the activity of IDH enzymes: Arg132 and Arg172 in IDH1 and Arg140 and Arg172 in IDH2 form hydrophilic hydrogen bonds with the α‐carboxyl and β‐carboxyl groups of isocitrate to facilitate isocitrate binding in the enzyme active site.^99^, ^100^ Mutations in these residues decrease binding affinity for isocitrate and increase binding affinity for NADPH, leading to the reduction of αKG by NADPH and the release of the (R) enantiomer of 2‐hydroxyglutarate [(R)‐2HG].^101^ The (R)‐2HG produced by the mutant IDH enzymes accumulates in cells to extremely high millimolar concentrations, disrupting a number of cellular functions.^100^ However, it is only the concentration and not the production of (R)‐2HG which is nonphysiological in *IDH*‐mutated tumors, as it is also produced as a minor product by αKG reductase enzymes and is oxidized back to αKG by 2HG dehydrogenases.^102^ Interestingly, cells expressing mutant IDH1 produce less (R)‐2HG than those with IDH2 mutations.^103^ Moreover, (R)‐2HG expression by mutant IDH1 is enhanced by the co‐expression of wild‐type IDH1, leading to the formation of heterodimers.^100^, ^103^ Loss of the wild‐type IDH1 expression results in a dramatic decrease in (R)‐2HG production,^104^ suggesting either that the presence of wild‐type IDH1 increases the enzymatic activity of mutant IDH1 toward αKG or that a cycling activity (redox neutral) permits a higher production of (R)‐2HG with a more stable cell phenotype.

Owing to the structural similarity of (R)‐2HG to αKG, the effect of *IDH* mutations on members of the αKG‐dependent dioxygenase family of enzymes has also been studied. Much like SDH‐ and FH‐mutated tumors, IDH‐mutated cells and tumors have been shown to exhibit a hypermethylator phenotype through the inhibition of the ten‐eleven translocation (TET) family of DNA demethylases and members of the Jumonji C‐domain histone demethylases through the competitive inhibition of αKG binding by (R)‐2HG.^105^, ^106^, ^107^, ^108^, ^109^ However, unlike *SDH* and *FH* mutations, the effect of (R)‐2HG on PHD activity has been the subject of some controversy. Although an initial report suggested that the PHD enzymes were inhibited by (R)‐2HG production resulting in increased HIF1α protein,^110^ the evidence that *IDH1* mutant cells and tumors actively suppress HIFα stabilization through activation of the PHDs is now more compelling. The precise means by which (R)‐2HG results in increased PHD enzyme activity, however, is still not clear. It has been suggested to be through direct use of only the (R) enantiomer by the PHDs as a substrate^109^, ^111^ or through nonenzymatic oxidation of 2HG into αKG, therefore acting as a source of the normal substrate.^112^ Consistent with either mechanism, IDH mutant cells display decreased HIF activation compared with their wild‐type counterparts.^109^ Mutations in *IDH1* and *IDH2*, in both glioma and AML, occur early in tumor development and are thought to be mutually exclusive,^52^, ^113^ suggesting that either isoform is sufficient to confer a predisposition to or permit transformation when mutated. The IDH1 and IDH2 isozymes differ only in their localization, yet although they are equally prevalent in patients with *IDH*‐mutated AML, gliomas with these mutations are almost always *IDH1*‐mutated. This discrepancy has not yet been explored fully, but it is possible that the environment in which each disease develops (such as the extent and severity of hypoxia) and/or a lack of suitable mitochondrial metabolic plasticity may play a role in determining why *IDH2* mutations are not observed often in gliomas.

Gliomas are highly hypoxic tumors.^114^, ^115^, ^116^ As outlined above, it has previously been shown that hypoxic cells may rely more on reductive glutamine metabolism to produce citrate (and thereby lipids) than normoxic cells.^30^ Both IDH1 and 2 have potential roles in hypoxia‐induced reductive carboxylation,^29^, ^31^ suggesting that this pathway may be perturbed in IDH1‐mutated gliomas. Given that the cytosolic pathway has been shown to be important in hypoxic reductive carboxylation for lipogenesis,^30^, ^32^, ^33^, ^34^ and that gliomas appear to ‘favor’ *IDH1* mutations, there may be a requirement for IDH2 wild‐type activity in hypoxic *IDH1* mutant gliomas. However, a recent paper also showed that *IDH1*‐mutated cells retained more of their oxidative TCA cycle metabolism than their wild‐type counterparts.^117^ Under hypoxic conditions, where *IDH1* wild‐type cells increased their dependence on reductive carboxylation of glutamine, *IDH1* mutant cells increased their use of oxidative decarboxylation, most likely retaining oxidative ATP generation in these conditions. This is consistent with the evidence that (R)‐2HG inhibits hypoxia‐induced stabilization of HIF1α,^109^ and therefore *IDH1*‐mutated cells may not exhibit the HIF‐dependent metabolic response to hypoxia, including changes in the activity of the PDC and complex IV. However, continued oxygen use in hypoxia is likely to create highly challenging conditions in an *IDH1*‐mutated tumor, as it is likely to further exacerbate the hypoxic microenvironment, potentially leading to increased areas of necrosis. It is important to note that *IDH1* mutant gliomas demonstrate improved overall survival compared to their wild‐type counterparts,^52^ which may well be in some part due to their apparent inability to modify their mitochondrial metabolism in response to hypoxia. Interestingly, *IDH2*‐mutated cells were shown not to exhibit the same alteration in the hypoxia‐mediated mitochondrial metabolic phenotype.^117^ These data may shed more light on the metabolic differences induced by *IDH1* and *IDH2* mutations and therefore their different prevalence in glioma—a highly hypoxic type of tumor.

## FUTURE PERSPECTIVES

It is thought that the ancestors of mitochondria, perhaps some type of Rhodobacteria, were organisms with a highly plastic metabolism, able to make ATP both in the presence and absence of oxygen. Although our mitochondria are removed from their predecessors by almost 2 billion years, the mutations that we observe in cancer and studies of other mammals have shown us that they retain many of these characteristics. It is also important to note that studies of mitochondrial metabolism are often centered around only one small aspect of their metabolism—the TCA cycle—and ignore other highly significant metabolic pathways for which mitochondria are critical. While classically being considered the TCA ‘cycle,’ it is becoming increasingly clear through studies on perturbed mitochondrial systems that it in fact consists of multiple interconnecting metabolic pathways that permit crosstalk between the mitochondrion and the cytosol. It is only through studies on tumors—highly proliferative cell systems—that we find that the cycle itself is dispensable, where the use of only some specific linear portions of the TCA cycle is required to fulfill the anabolic requirements without significant difficulty. However, it is in adapting to the truncated nature of the TCA cycle that *SDH*‐, *FH*‐, and *IDH*‐mutated and hypoxic cells have been shown to become highly dependent on one or more pathways for their survival. These metabolic vulnerabilities therefore represent attractive targets for future therapeutic development.
